# Predator community and resource use jointly modulate the inducible defense response in body height of crucian carp

**DOI:** 10.1002/ece3.7176

**Published:** 2021-02-03

**Authors:** Ilaria de Meo, Kjartan Østbye, Kimmo K. Kahilainen, Brian Hayden, Christian H. H. Fagertun, Antonio B. S. Poléo

**Affiliations:** ^1^ Department of Forestry and Wildlife Management Inland Norway University of Applied Sciences Koppang Norway; ^2^ Lammi Biological Station University of Helsinki Helsinki Finland; ^3^ Biology Department Canadian Rivers Institute University of New Brunswick Fredericton NB Canada

**Keywords:** body shape, lake productivity, phenotypic plasticity, predation risk regime, resource use

## Abstract

Phenotypic plasticity can be expressed as changes in body shape in response to environmental variability. Crucian carp (*Carassius carassius*), a widespread cyprinid, displays remarkable plasticity in body morphology and increases body depth when exposed to cues from predators, suggesting the triggering of an antipredator defense mechanism. However, these morphological changes could also be related to resource use and foraging behavior, as an indirect effect of predator presence. In order to determine whether phenotypic plasticity in crucian carp is driven by a direct or indirect response to predation threat, we compared twelve fish communities inhabiting small lakes in southeast Norway grouped by four categories of predation regimes: no predator fish, or brown trout (*Salmo trutta*), perch (*Perca fluviatilis*), or pike (*Esox lucius*) as main piscivores. We predicted the body shape of crucian carp to be associated with the species composition of predator communities and that the presence of efficient piscivores would result in a deeper body shape. We use stable isotope analyses to test whether this variation in body shape was related to a shift in individual resource use—that is, littoral rather than pelagic resource use would favor the development of a specific body shape—or other environmental characteristics. The results showed that increasingly efficient predator communities induced progressively deeper body shape, larger body size, and lower population densities. Predator maximum gape size and individual trophic position were the best variables explaining crucian carp variation in body depth among predation categories, while littoral resource use did not have a clear effect. The gradient in predation pressure also corresponded to a shift in lake productivity. These results indicate that crucian carp have a fine‐tuned morphological defense mechanism against predation risk, triggered by the combined effect of predator presence and resource availability.

## Graphical abstract

The crucian carp develops a deep body when exposed to cues from predators and represents a classic example of anti‐predator defense mechanism in fish. In this study, we showed that crucian carp body depth increased with decreasing trophic position and increasing predation risk represented by predator gape size. Our results indicate that crucian carp have a fine‐tuned morphological defense mechanism against predation risk, triggered by the combined effect of predator community and resource use.

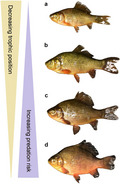

## 1 INTRODUCTION

Phenotypic plasticity is the ability of an organism to express different phenotypes in response to environmental variation (Pigliucci, 2001). Plastic responses can be a successful strategy in spatially or temporally heterogeneous environments, where organisms can improve their fitness by adjusting morphological, physiological or behavioral traits in relation to different abiotic and biotic conditions (Gabriel, 2005; Lind & Johansson, 2007; Miner et al., 2005). However, the ecological and evolutionary significance of phenotypic plasticity is still under debate (Pfennig et al., 2010; Price et al., 2003; Uller et al., 2019). Phenotypic plasticity can facilitate adaptation to novel environments, allowing populations to occupy different ecological niches that may lead to speciation events (Corl et al., 2018; Skúlason et al., 2019). At the same time, its benefits could be constrained by the energetic costs associated with the production and maintenance of plastic responses as well as limits in the predictability and reliability of environmental cues (DeWitt et al., 1998; Snell‐Rood et al., 2010).

Phenotypic plasticity can be expressed as variation in body shape in response to interactions with other species, different resource availability, as well as different habitat characteristics. Many freshwater organisms can adopt predator‐induced morphological defenses when exposed to a predation threat (Bourdeau & Johansson, 2012; DeWitt et al., 2000; Sperfeld et al., 2020). Here, chemical cues from predators or injured conspecifics induce a morphological change in the prey that make them less vulnerable to predation (Harvell, 1990). For example, in the presence of predators, pumpkinseed sunfish (*Lepomis gibbosus*) increases its defensive structure such as dorsal spine length and body depth (Januszkiewicz & Robinson, 2007). Another classic example of inducible antipredator defense mechanism in fish is the crucian carp (*Carassius carassius*), which develops a deep body when exposed to cues from predators such as perch (*Perca fluviatilis*) or pike (*Esox lucius*) (Brönmark & Pettersson, 1994). Flexibility in prey morphological and behavioral responses might be a widespread strategy, given that species composition of predators often varies greatly among locations and over time (Kishida & Nishimura, 2006). Indeed, although with consistent differences among species, variation in body shape in response to predator presence has been hypothesized in various freshwater fish such as perch, roach (*Rutilus rutilus*), three‐spined sticklebacks (*Gasterosteus aculeatus*), and fathead minnow (*Pimephales promelas*) (Eklöv & Jonsson, 2007; Frommen et al., 2011; Meuthen et al., 2019).

In general, predators play an important role in structuring freshwater ecosystems. Different predators can influence prey dynamics and select specific morphological and behavioral traits of prey by variation in their density, gape size and foraging strategies (Magnhagen & Heibo, 2004; Scharf et al., 2000; Sharma & Borgstrøm, 2008). For example, pike is a sit‐and‐wait predator, attacking from littoral vegetation (Skov & Nilsson, 2018; Turesson & Brönmark, 2004) and tends to prey upon nonvigilant individuals (Heynen et al., 2017). In contrast, piscivorous perch hunt actively for prey and select mainly mobile, bold individuals (Heynen et al., 2017). Piscivorous fish such as pike are also gape‐size limited in their prey selection and often prefer to select shallow‐bodied individuals, since handling time increases with prey body depth (Nilsson et al., 1995). Selective consumption can cause a shift in the phenotypic distribution of prey, since large deep‐bodied individuals which are outside the predation window are more likely to survive (Nilsson & Brönmark, 2000). In turn, this shift can have indirect effects that influence dramatically prey competitive interactions and community dynamics (Peacor & Werner, 2001; Siepielski et al., 2020).

Moreover, the role of resource use in predator‐induced morphological defenses has been recently debated, since trade‐offs occur among predation risk and resource acquisition (Scharnweber et al., 2013; Svanbäck et al., 2017). In this sense, lake morphology and water quality regulate availability and quality of food resources that, in turn, influence both population density and individual growth rate (Horppila et al., 2010). In particular, fish condition generally increases with lake productivity since nutrients fuel the base of the food web, increasing available resources for consumers (Weber et al., 2010). At the same time, food acquisition and growth rate are often highly influenced by intraspecific competition and thus negatively related to population density (Amundsen et al., 2007; Svanbäck & Persson, 2004). Predation can also indirectly induce a change in prey morphology causing shifts to habitat with different food quality (Preisser et al., 2005). In this case, an alteration in prey phenotype can represent a foraging adaptation that promotes specialization in acquiring specific resources in the new habitat (Ellerby & Gerry, 2011). Thus, it is pertinent to address the question whether the predator cues alone result in morphological change in the prey, or if the changed foraging habitat of the prey is driving the altered morphology as a secondary response. Alternatively, and more likely, evolutionary optimization of the trade‐off regime may result from both selective pressures jointly.

In this study, we examine crucian carp body shape and depth from lakes and ponds with different piscivore assemblages and environmental characteristics, testing also for associations between predator‐induced shifts in resource use and morphology. Because of its unique physiological adaptations, crucian carp is often the only fish species able to survive in anoxic waters of shallow ponds during winter (Blažka, 1958; Piironen & Holopainen, 1986). High densities of small‐sized and shallow‐bodied fish characterize populations occurring in these ponds, where obvious resource limitation leads to strong intraspecific competition (Pettersson & Brönmark, 1997). On the other hand, multispecies assemblages in larger lakes contain low densities of deep‐bodied crucian carp, for which predation is likely the main regulating force (Poléo et al., 1995). In these lakes, a deep body represents a morphological defense against gape‐limited predators (Nilsson & Brönmark, 2000). However, experiments have shown that enhanced food availability and low population densities alone can cause a similar increase in relative body depth compared to predation risk (Holopainen et al., 1997; Tonn et al., 1994), suggesting that growth and morphology are also dependent on resource availability. Moreover, in a manipulative experiment, Andersson et al., (2006) observed that crucian carp feeding on benthic prey rather than zooplankton developed a deeper body, similarly to the fish exposed to cues from predators. With an analogous experimental approach, it was found that both standing water conditions and exposure to predation cues independently induced a deeper body in crucian carp (Johansson & Andersson, 2009). Thus, it has been proposed that this increase in body depth could be associated with an alteration in foraging behavior and activity levels of the fish, suggesting that more complex mechanisms may control the morphology of this species than the sole predation risk (Vøllestad et al., 2004). Laboratory experiments also show that crucian carp habitat use was significantly affected by both predation risk and hunger level, indicating a trade‐off between food acquisition and predator avoidance (Pettersson & Brönmark, 1993). Moreover, in presence of predators, the structural complexity offered by vegetation of near shore habitats may enhance the chance of survival of crucian carp until they reach a certain body depth (Holopainen et al., 1997). In this environment, benthic invertebrates associated with the substrate or vegetation are the most abundant prey type and a deep body might provide fish with a greater maneuverability and foraging efficiency (Svanbäck & Eklöv, 2003; Webb, 1984). In contrast, if predation pressure is released, fish would rely more on pelagic invertebrates and show a slender body shape. Hence, discerning how different environmental factors affect plastic responses in crucian carp may help us gain a better understanding of their evolutionary and ecological significance for freshwater fish.

Here, we examined crucian carp body shape and trophic niche variability by landmark‐based geometric morphometrics and stable isotope analysis (SIA) in a series of small lakes. We used stable isotopes of carbon and nitrogen to estimate the trophic position and relative contribution of littoral and pelagic energy to each crucian carp sampled in each lake. Individual trophic specialization could reveal potential variation in crucian carp habitat preferences and resource use, which could be reflected in specific body morphology. Thus, a more extensive morphometric analysis of wild populations of this species could potentially show variation in different traits other than body depth. This approach differs from previous experimental studies (Andersson et al., 2006; Johansson & Andersson, 2009; Vøllestad et al., 2004), as we consider a comprehensive set of abiotic and biotic characteristics that might directly or indirectly underlie crucian carp body development. Moreover, we included locations with multiple predators to quantify the predation risk associated with each lake. Here, a set of three replicate lakes of four categories of predation regimes was tested, being allopatric lakes (no predators), and sympatric lakes with three increasingly efficient main predators: brown trout (*Salmo trutta*), perch, and pike. Brown trout and perch are opportunistic piscivores. In small lakes, invertebrates are the major food source of brown trout and perch until they shift to a diet mainly consisting of fish, and this switch to piscivory generally occurs at a larger size for trout (Mittelbach & Persson, 1998). On the contrary, pike is a specialist piscivore throughout its life and grows to large body and gape size, representing an efficient predator that can impose a greater risk for a broad range of prey size classes (Mittelbach & Persson, 1998).

In particular, in the present study, we expected the body shape of crucian carp to be associated with the species composition of predators in the lakes investigated and thus that increasingly efficient predators would cause progressively deeper body shape. We also wanted to evaluate if such variation in body shape depended on predator‐induced shifts in habitat and resource use. Specifically, we wanted to test whether crucian carp under increasing predation risk feed more on littoral resources associated with substrate or vegetation, compared to crucian carp in lakes without predators (Pettersson & Brönmark, 1993). Moreover, we predicted that variation in body shape was modulated by the synergistic effect of predation risk (predator mouth gape and density) and specific abiotic factors (lake morphology and productivity).

## 2 METHODS

### 2.1 Study area and sample collection

**FIGURE 1:**
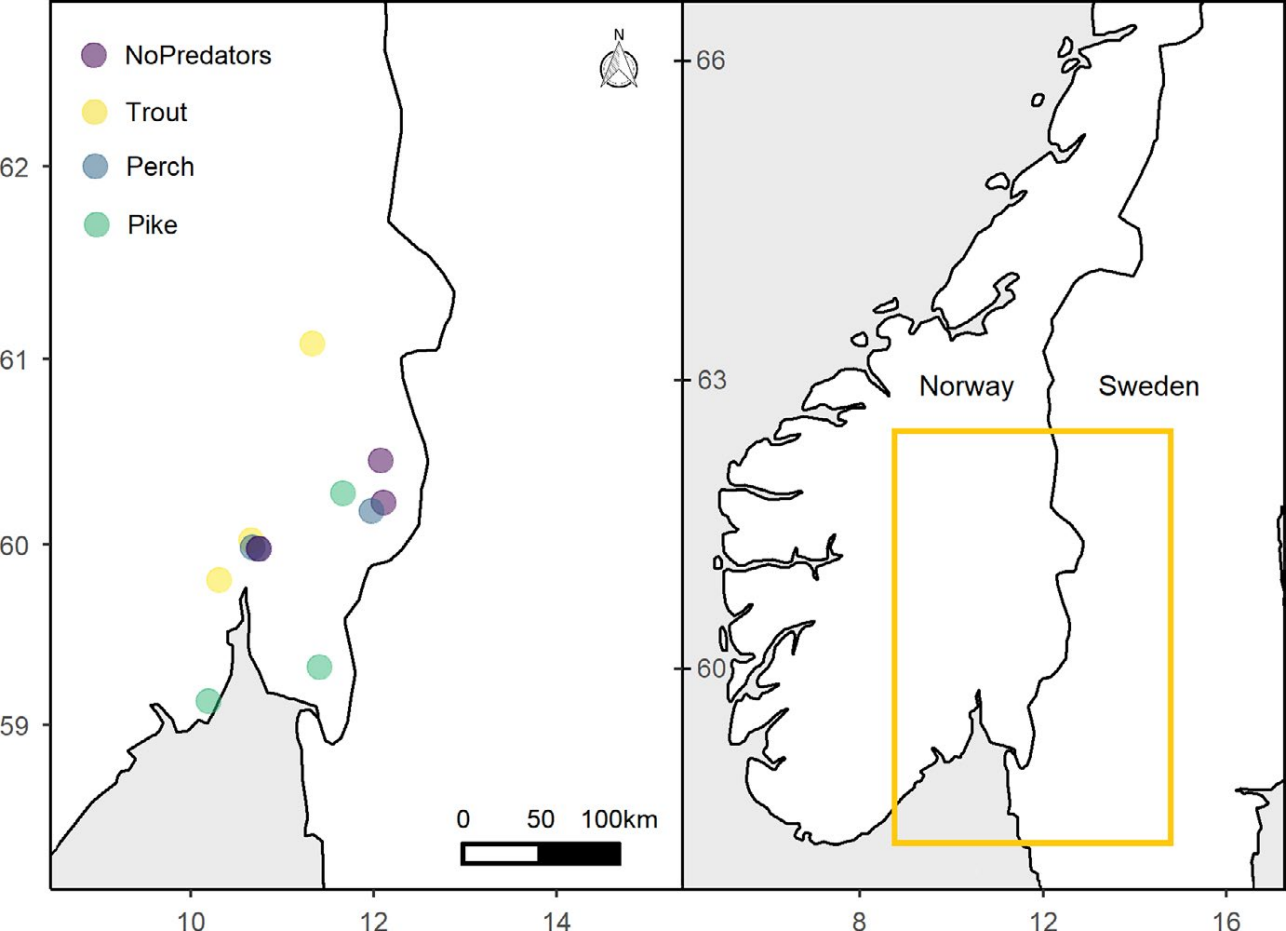
Location of the twelve sampling sites in southeastern Norway

We sampled twelve fish communities from small (0.25–11 ha) and relatively shallow (max depth 1.5–11.3 m) lakes located in southeastern Norway between June and August in 2018 and 2019 (Figure 1; Table 1). All lakes were characterized by high densities of macrophytes. Abiotic parameters collected include lake surface area (ha), maximum depth (m), and nutrient concentration (Table 1). We estimated lake bathymetry in the field with a portable echosounder. Total nitrogen (µg/l), phosphorus (µg/l), and organic carbon (mg/l) were determined from surface water samples for ten lakes and retrieved from the Norwegian Environment Agency for two lakes (vannmiljofaktaark.miljodirektoratet.no).

**TABLE 1:** Environmental characteristics of the study lakes

Lake	Lat (°N)	Long (°E)	Alt (m a.s.l.)	Area (ha)	MaxD (m)	TotN (µg/l)	TotP (µg/l)	TOC (mg/l)	Fish species
Bugårdsdammen	59.13	10.2	42	5.04	2	980	54	9.5	a, b, c
Stomperudtjernet	59.32	11.4	103	3.85	1.5	1,660	146	18.4	a, b, c, e, f, g
Nusttjennet	60.28	11.66	131	11.00	1.5	1,090	164	16.4	a, b, c, e, f
Øvresetertjern	59.98	10.67	478	3.05	3.5	446	13	6.6	a, c, d
Svartkulp	59.98	10.74	202	5.80	10	550	13	9.9	a, c, d, h
Bjørnmyrdammen	60.18	11.98	256	2.10	3.5	672	26	6.5	a, c, i
Posttjernet	61.08	11.33	271	1.72	11	312	8	9.7	a, d, h
Småvanna	59.8	10.31	222	0.50	3.8	616	14	10.1	a, d, h
Karussputten	60.02	10.66	356	0.25	4.6	361	9	5.4	a, d
Forkerudstjennet	60.45	12.08	152	1.24	2.2	1,985	82	23.4	a
Langmyrtjern	59.97	10.75	206	0.30	5	702	20	14.2	a, h
Motjennet	60.23	12.11	167	0.94	11.3	688	23	11.2	a

Note

Variables include latitude (Lat), longitude (Long), altitude (Alt), lake area (Area), maximum depth (MaxD), total nitrogen (TotN), total phosphorus (TotP), total organic carbon (TOC), and fish species present.

Fish species: (a) crucian carp; (b) pike; (c) perch; (d) brown trout; (e) roach *Rutilus rutilus*; (f) bream *Abramis brama*; (g) rudd *Scardinius erythrophthalmus*; (h) minnow *Phoxinus phoxinus*; (i) tench *Tinca tinca*.

Locations were chosen along a gradient of predation pressure. We grouped lakes into four categories according to species composition of predators in the systems: no predators, brown trout (hereafter trout), perch, and pike lakes (Tables 2 and 3). No predators occurred in Forkerudtjern, Langmyrtjern, and Motjennet. Brown trout was the only predator in Karussputten, Småvanna, and Posttjernet. Perch was the main predator in Svartkulp, Bjørnmyradammen, and Øvreseterjern while trout was present with very low density or absent. Both perch and pike were present in Bugårdsdammen, Stomperudtjernet, and Nusttjennet, but we will refer to these lakes as “pike lakes” for simplicity. We assessed fish density in each lake using Nordic multimesh gillnets consisting of 12 equidistant panels (mesh sizes 5–55 mm) and calculated CPUE (*n* fish·net^−1^ h^−1^) for littoral, profundal, and pelagic habitats. Then, proportions of predators and crucian carp were calculated as the respective densities from CPUE data relative to the total fish present in each lake (Table 3). Some lakes had very limited pelagic and profundal habitats and were considered as entirely littoral. Moreover, we did not catch any fish in the profundal zone, probably because deeper lakes were highly humic systems with hypoxic deep waters. Consequently, fish density and biomass analysis were limited to the littoral and pelagic zones. We also used a variety of fishing methods (e.g., baited traps, gillnets with different mesh sizes, kick nets) to increase our catch of small crucian carp, since these fish often display an elusive behavior and alter diel activity patterns when occurring with predators (Vinterstare et al., 2020). Immediately after capture, fish were euthanized by an overdose of tricainemethanesulfonate (MS222) and transported to the laboratory. Permission to catch fish was given by the Norwegian Environmental Agency (2018/4155) and fish were sampled after oral approval by the local landowners.

**TABLE 2:** Mean and standard deviation of total length (TL), body height (BH), carbon (δ13C) and nitrogen (δ15N) stable isotopes, littoral reliance (LIT), trophic position (TP), and sex ratio of crucian carp

Lake	Predation category	TL (cm)	BH (cm)	δ^13^C (‰)	δ^15^N (‰)	LIT	TP	Sex ratio (m/f)
*Bugårdsdammen*	Pike	31.5 ± 6.8	12.4 ± 2.4	−30.2 ± 0.7	8.7 ± 0.7	0.5 ± 0.3	1.9 ± 0.2	6.5
*Stomperudtjernet*	Pike	19.1 ± 9.5	7.7 ± 3.9	−32.2 ± 0.7	13.6 ± 1.2	0.9 ± 0.4	1.9 ± 0.3	4
*Nusttjennet*	Pike	33.1 ± 1.6	13.5 ± 0.5	−31.6 ± 0.3	10.6 ± 0.4	0.8 ± 0.0	1.8 ± 0.1	1.3
*Øvresetertjern*	Perch	28.8 ± 3.6	10.6 ± 1.4	−28.1 ± 0.5	7.0 ± 0.4	0.8 ± 0.4	2.2 ± 0.1	1.3
*Svartkulp*	Perch	19.8 ± 4.7	6.5 ± 1.6	−32.3 ± 0.7	5.6 ± 0.4	0.9 ± 0.2	2.0 ± 0.1	2
*Bjørnmyrdammen*	Perch	18.0 ± 1.7	6.0 ± 06	−32.2 ± 0.8	6.0 ± 0.4	0.6 ± 0.1	2.3 ± 0.1	1.5
*Posttjernet*	Trout	19.3 ± 3.6	6.9 ± 1.5	−34.1 ± 1.3	5.7 ± 0.3	0.5 ± 0.3	2.3 ± 0.1	1.7
*Småvanna*	Trout	15.4 ± 2.2	4.9 ± 0.7	−36.5 ± 1.4	8.6 ± 0.7	0.4 ± 0.3	2.0 ± 0.2	1.4
*Karussputten*	Trout	14.5 ± 3.4	4.5 ± 1.1	−35.0 ± 1.2	3.6 ± 0.4	0.1 ± 0.3	1.5 ± 0.1	1
*Forkerudstjennet*	No pred.	11.4 ± 1.9	3.3 ± 0.7	−31.9 ± 1.0	10.1 ± 0.9	0.8 ± 0.2	2.2 ± 0.2	0.2
*Langmyrtjern*	No pred.	10.7 ± 2.8	2.8 ± 0.9	−34.5 ± 0.9	4.5 ± 0.6	0.5 ± 0.3	1.7 ± 0.2	0.3
*Motjennet*	No pred.	11.9 ± 3.0	3.4 ± 0.9	−32.7 ± 1.4	5.4 ± 0.7	0.6 ± 0.2	2.1 ± 0.2	1

**TABLE 3:** Predator species present in each lake, number of pike, perch and trout measured in the laboratory, mean and standard deviation of maximum predator gape size (MaxGS*, n = *10), predator density (CPUEpred) and proportion (RelPred), and crucian carp density (CPUEcc) and proportion (RelCc)

Lake	Predator species	No. Pike	No. Perch	No. Trout	MaxGS (mm)	CPUEpred (n.net^−1^ h^−1^)	RelPred (%)	CPUEcc (n.net^−1^ h^−1^)	RelCc (%)
*Bugårdsdammen*	Pike, perch	27	337	—	61.0 ± 14.2	1.6 ± 0.6	93.8	0.1 ± 0.1	6.2
*Stomperudtjernet*	Pike, perch	7	25	—	56.2 ± 26.0	0.4 ± 0.0	5.0	0.4 ± 0.2	5.7
*Nusttjennet*	Pike, perch	27	24	—	47.3 ± 11.9	0.3 ± 0.3	4.6	0.4 ± 0.2	6.2
*Øvresetertjern*	Perch, trout	—	286	36	44.3 ± 7.0	3.6 ± 1.9	89.4	0.4 ± 0.6	10.6
*Svartkulp*	Perch, trout	—	151	7	34.8 ± 4.4	2.1 ± 1.3	68.9	0.4 ± 0.4	12.2
*Bjørnmyrdammen*	Perch	—	34	—	26.7 ± 2.1	0.4 ± 0.3	16.1	2.1 ± 2.1	83.2
*Posttjernet*	Trout	—	—	89	37.9 ± 1.7	0.9 ± 1.1	53.3	0.2 ± 0.2	10.6
*Småvanna*	Trout	—	—	17	33.1 ± 7.0	0.3 ± 0.4	18.0	1.0 ± 1.1	60.8
*Karussputten*	Trout	—	—	12	32.8 ± 4.8	0.2 ± 0.2	13.8	1.0 ± 0.2	86.2
*Forkerudstjennet*	—	—	—	—	—	—	—	10.6 ± 5.9	100.0
*Langmyrtjern*	—	—	—	—	—	—	—	2.1 ± 1.8	77.0
*Motjennet*	—	—	—	—	—	—	—	7.8 ± 4.4	100.0

In order to estimate the basal resources for stable isotope analysis (SIA), we collected qualitative samples of benthic invertebrates and zooplankton. We sampled benthic invertebrates from sediments and plants in the littoral habitat using kick nets and sorted them to the lowest feasible taxonomic level. We collected zooplankton from several hauls through the water column in the pelagic zone of deep lakes and in the nonvegetated area of shallow lakes with a 50‐μm mesh plankton net. Samples were later sieved through a 200‐μm mesh to remove unwanted material. The remaining zooplankton were identified to class level.

### 2.2 Laboratory analysis

Body shape was measured from a total of 360 crucian carp. From each lake, we subsampled 30 crucian carp for morphometric analysis (Table 2). In general, fish size structure was quite uniform within each lake and dominated by large individuals. Thus, we included smaller crucian carp whenever possible to avoid underrepresentation of this size class. From the same fish, a piece of dorsal muscle tissue was dissected and frozen at −20°C for SIA. We also measured length and gape height of the most abundant piscivorous fish, that is, trout, perch, and pike (Table 3). Mouth height was measured as the maximum distance between the tip of the premaxilla and the mandible with the mouth stretched open. Then, we calculated the mean maximum gape size from each lake by selecting the predators with the highest mouth height, irrespective of the species (*n* = 10).

#### 2.2.1 Morphometric analysis

**FIGURE 2:**
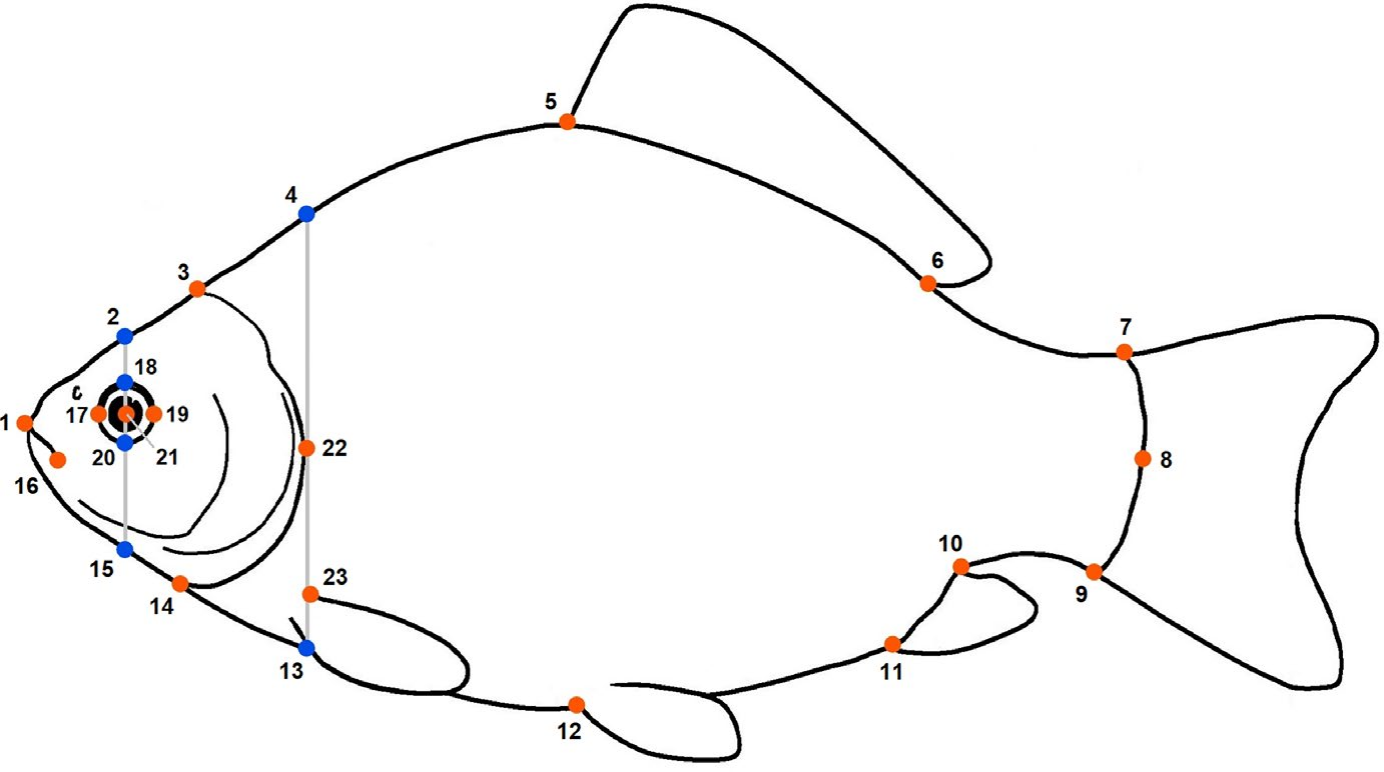
Crucian carp line drawing showing the location of 17 landmarks (red dots) and 6 semilandmarks (blue dots) used in geometric morphometric analysis. Homologous landmarks (red dots) indicate tip of the snout (1), posterior dorsal margin of the head (3), anterior insertion of dorsal fin (5), posterior insertion of dorsal fin (6), dorsal insertion of caudal fin (7), posterior margin of caudal peduncle (8), ventral insertion of caudal fin (9), posterior insertion of caudal fin (10), anterior insertion of caudal fin (11), insertion of pelvic fin (12), posterior ventral margin of the head (14), posterior margin of mouth (16), anterior margin of eye (17), posterior margin of eye (19), center of eye (21), posterior margin of operculum (22), dorsal insertion of pectoral fin (23). Semilandmarks placed along axis passing through the center of eye and the posterior edge of the operculum indicate dorsal midpoint of head (2), anterior dorsal midpoint of body (4), anterior ventral midpoint of body (13), ventral midpoint of head (15), dorsal margin of eye (18), and ventral margin of eye (20). Picture adapted from the Crucian Carp Field Identification Guide by the UK Environment Agency (www.gov.uk/environment‐agency)

We examined crucian carp body shape using landmark‐based geometric morphometrics. We laterally photographed fish using a Nikon D5300 camera positioned on a tripod and set at a focal length of around 60 mm. In order to minimize perspective and distortions errors among images, we arranged fish along their main horizontal axis, extended dorsal and ventral fins using dissecting pins, and used a mesh cradle (Muir et al., 2012). Digital photographs were transferred to tpsDig2 software v 2.31 (Rohlf, 2004), and 17 landmarks and six semilandmarks were digitized (Figure 2). Digitizing was always performed by the same person. After checking for outliers, we used a Generalized Procrustes Analysis (GPA) to standardize the landmark configurations for position, orientation, and size. Centroid size (CS) of the landmark configurations was used as a proxy for body size. Centroid size is the square root of the sum of the squared distances of landmarks from their center of gravity (centroid). Centroid size values were log‐transformed prior to statistical analysis. All morphometric analysis was performed using the package “Geomorph” (Adams et al., 2020) in R version 4.0.1 (R Core Team, 2020).

#### 2.2.2 Stable isotope analysis

Fish muscle and invertebrate samples were freeze‐dried at −50°C for 48 hr, ground to a homogeneous powder, weighed (1.0–1.2 mg), and encapsulated into tin cups. Stable carbon and nitrogen isotope ratios were analyzed by a Costech 4010 elemental analyzer (Costech) coupled to a Delta Plus continuous flow mass spectrometer (Thermo Finnigan). Stable isotope measurements are expressed as *δ* ^13^C and *δ* ^15^N in parts per thousand (‰) relative to the international standards Vienna Pee Dee Belemnite and atmospheric air for carbon and nitrogen, respectively. Standard deviation of internal working standards was less than 0.1‰ for *δ* ^13^C and 0.2‰ for *δ* ^15^N. C:N ratios from fish data were low in all samples (3.3 ± 0.1) indicating low lipid concentrations (Fagan et al., 2011; Kiljunen et al., 2006). Thus, we did not lipid‐correct *δ* ^13^C ratios. Since basal resource values can vary greatly among different systems, we standardized crucian carp *δ* ^13^C and *δ* ^15^N ratios by using littoral and pelagic invertebrates in each lake as baseline. Individual trophic position and littoral reliance (i.e., relative contribution of littoral prey items to crucian carp diet) were calculated using a two‐source mixing model (Karlsson & Byström, 2005) with trophic fractionation values of 3.4‰ for *δ* ^15^N and 0.4‰ for *δ* ^13^C (Post, 2002).

### 2.3 Data analyses

#### 2.3.1 Body shape analysis

Principal component analysis (PCA) on Procrustes shape coordinates was used to identify the major patterns of shape variation and grouping of variance among individuals. Thin‐plate deformation grids were used to visualize variation at the lowest and highest values along the first principal component axis. In order to investigate variation of crucian carp body shape among predation categories (no predators, trout, perch, and pike), principal component scores were examined through Discriminant Function Analysis (DFA) in the R package “MASS.” The maximum number of principal components to retain in the analysis was estimated by the broken stick model. Validity of discrimination was tested by jackknifed cross‐validation. A Procrustes ANOVA with permutation procedures was used to estimate allometric effects (i.e., shape variation in relation to size) among predation categories in the R package “Geomorph” (Adams et al., 2020). Procrustes shape coordinates were used as response variables, log‐transformed centroid size as predictor variable and predation as categorical variable with lake as nested effect. Since allometry had a significant effect on shape, centroid size was used as a covariate in subsequent linear models. Shape differences between sexes were significantly different but explained only a very small part of variation (*R* ^2^: 0.047, *p*‐value: 0.001). Males had slightly larger dorsal region than females; however, females alone expressed the same changes along the PC axes, indicating a minor effect of sex. Therefore, females and males were pooled in the analysis.

#### 2.3.2 Association of body depth with environmental variables

We determined if crucian carp assemblages occupied distinct isotopic niches using a permutational multivariate analysis of variance (PERMANOVA; Anderson, 2001) of a Euclidean distance matrix of littoral reliance and trophic position. Predation category and Lake were used as factors in the analysis. In addition, we used a distance‐based test for homogeneity of multivariate dispersions (PERMDISP; Anderson, 2006) to evaluate differences in within‐group variability of Lakes and Predation factors. Analysis was performed in R using the *adonis* and *betadisper* functions in the “vegan” package (Oksanen et al., 2019). We used linear mixed‐effects models (LME) to examine the degree of relationship between variation in body depth and specific biotic and abiotic characteristics associated with each lake. The scores of the first axis of principal component (PC1), which corresponded largely to the fish body depth, were used as the response variable. More precisely, considering that Procrustes superimposition controls the size effects through scaling, the response variable represents crucian carp relative body depth. Candidate explanatory variables for predation risk included predation category (Pred), maximum predator gape size (MaxGS), predator density (CPUEPred), and predator proportion (RelPred). Density (CPUECc) and proportion (RelCc) of crucian carp were used as a proxy for intraspecific competition. However, predator density and proportion were positively correlated (*r* > 0.8), and only the latter was included in the final model. Moreover, both crucian carp density and proportion were excluded, since negatively correlated with the maximum predator gape size (*r* < −0.8). Littoral reliance (LIT) and trophic position (TP) were used as a measure of individual crucian carp resource use. Abiotic characteristics included lake area, maximum depth (MaxD), and total nutrients. Among nutrient variables, only total phosphorus (TotP) was used in the analysis, since it was positively correlated with both total nitrogen and organic carbon (*r* ≥ 0.7). The full model takes the form:

Body depth ~ RelPred + MaxGS + LIT + TP + MaxD + TotP + Area + logCsize.

Model selection was performed by stepwise selection based on the Akaike information criterion (AIC). Lakes were used as a random factor nested in the predation category term. Model assumptions of normality and homogeneity of residuals were met and validated using a QQ‐plot and plotting residuals against fitted values, respectively. Correlation between variables was tested using the *ggpairs* function in the “GGally” package (Emerson et al., 2013). Analyses were performed in R using “lme4” and “lmerTest” packages (Bates et al., 2015).

## 3 RESULTS

### 3.1 Body shape analysis

**FIGURE 3:**
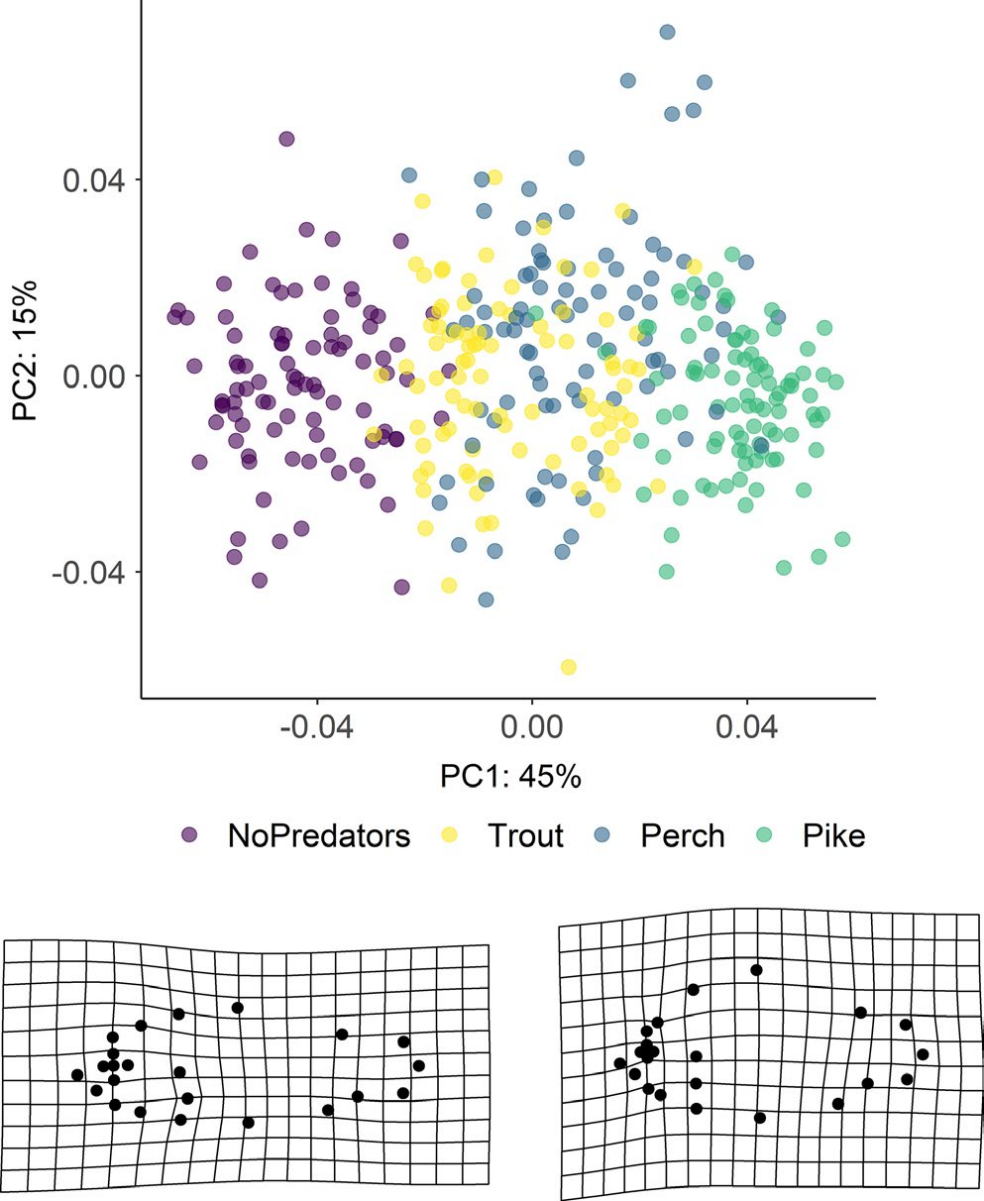
Scatterplot of principal components analysis of body shape of crucian carp. Individuals are color‐coded according to predation category. Deformation grids show the most extreme negative and positive shapes along the first (PC1) axis. Percentages indicate how much of the variation is explained by the first two axes

**FIGURE 4:**
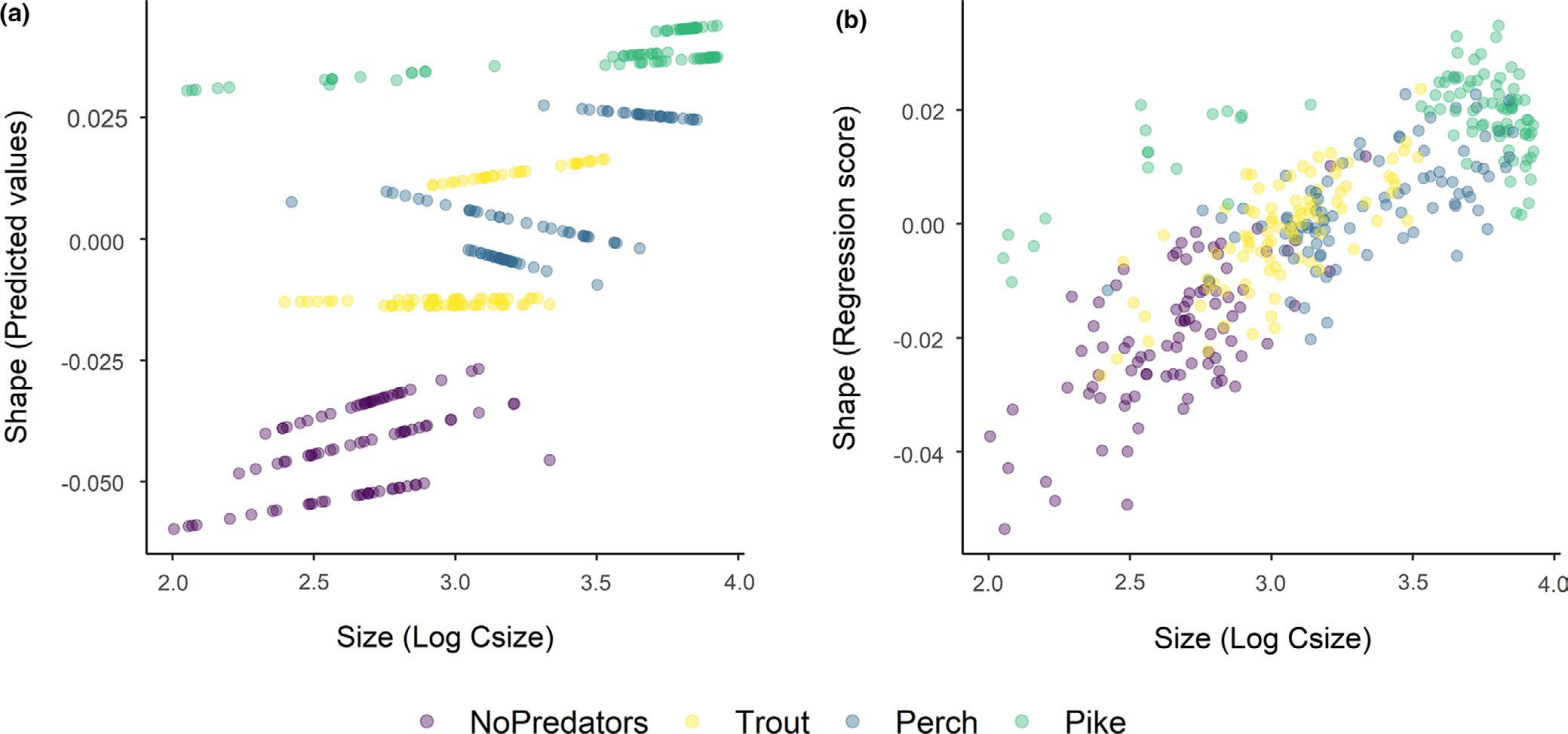
Allometric trajectories of crucian carp from twelve lakes with different predation regimes calculated using the *plotAllometry* function in the R package “Geomorph” (Adams et al., 2020). The x‐axis values represent the log‐transformed centroid size (LogCsize) as a proxy for individual body size. The y‐axis values represent a) the shape as the first principal component of the predicted values and b) the standardized shape scores from the multivariate regression of shape on size

The first three axes of the PCA of landmark configurations (Figure 3) accounted for 67% of the variation in body shape, with PC1, PC2, and PC3 explaining 45%, 15%, and 7% of the total variance, respectively. Shape variation along the PC1 axis was mainly associated with the expansion of the dorsal (landmarks 4, 5, 6) and ventral (landmarks 11, 12) regions, indicating an overall change in body depth (see Table S1 in Appendix S1). PC2 described mainly variation in body curvature, with snout (landmarks 1, 16) and caudal peduncle (landmarks 6, 7, 8) bending slightly downwards, and ventral and dorsal parts (landmarks 4, 5, 12) shifting upwards, indicating an overall flattering of the ventral region along the axis. PC3 explained variation in head size (landmarks 1, 22), body slenderness (landmarks 4, 5, 7, 8, 11, 12, 13), and insertion of the pectoral fin (landmark 23). Fish grouped along the first PC axis mainly according to the different predation categories. In absence of predators, fish had a slender body shape, which got increasingly rounded in presence of trout, perch, and pike. The same grouping was revealed by DFA as complementary method (Figure S1). DFA produced three significant DF axes differentiating between predation categories, and DFA1, DFA2, and DFA3 accounted for 92.7%, 7.2%, and 0.1% of shape variation, respectively. Jackknifed validation indicated that 79% of crucian carp were assigned to the correct predation category (Table S2). Individuals from “No predators” and “Pike” groups were generally correctly classified (≥90%), while individuals from “Trout” and “Perch” groups were more frequently classified as each other. Results of Procrustes ANOVA (Table S3) show that the body shape of crucian carp was positively related to the logarithm of centroid size (*F*: 191.42, *p*‐value: 0.001), predation category (*F*: 46.41, *p*‐value: 0.001) and their interaction with (*F*: 6.89, *p*‐value: 0.001) and without lake effect (*F*: 7.19, *p*‐value: 0.001). The significance of the interaction terms suggests nonparallel slopes and thus that shape variation in relation to size differs among predation categories—for example, small‐sized fish from pike lakes have deeper body depth than fish of the same size from lakes without predators (Figure 4).

### 3.2 Major variables explaining variation in body depth

**FIGURE 5:**
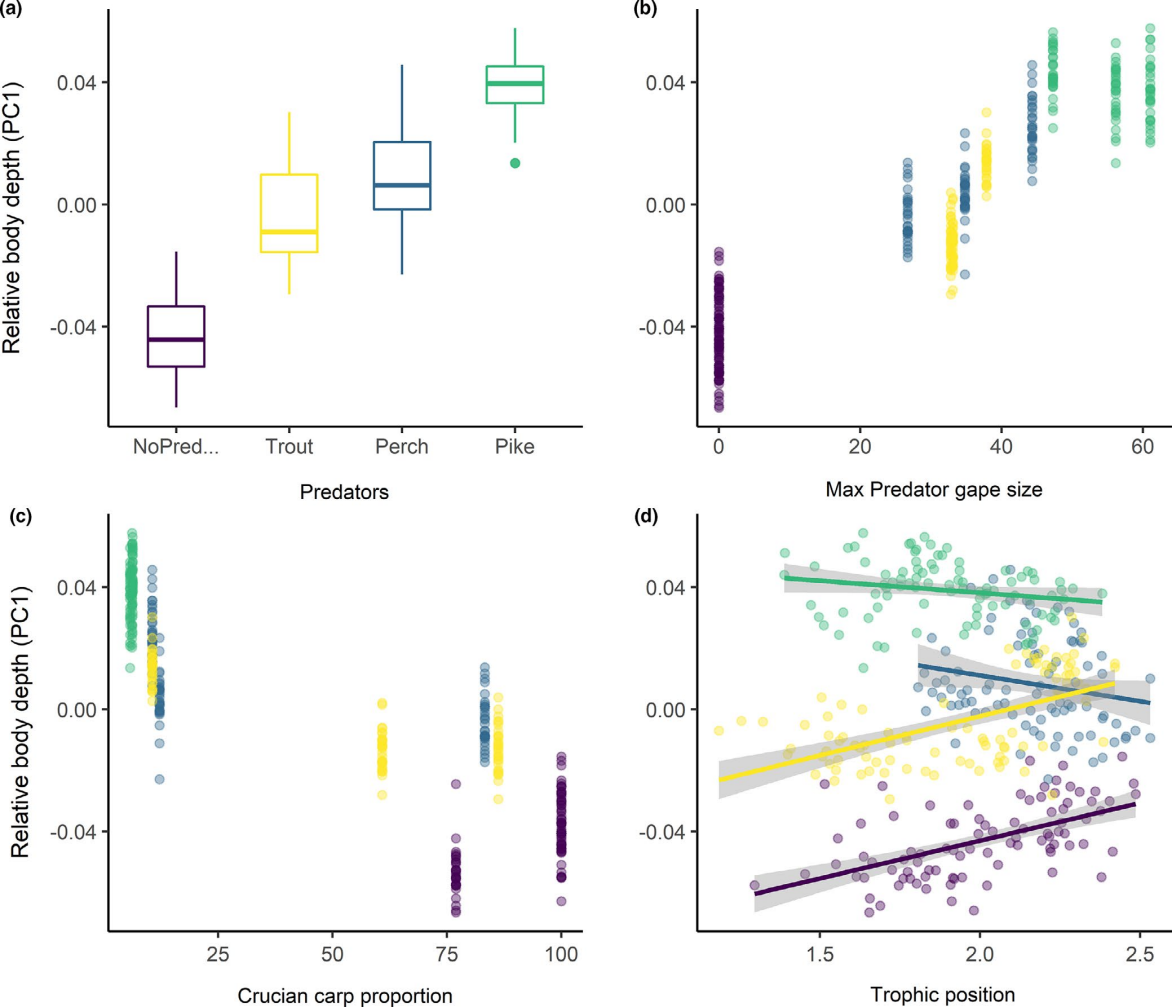
Relationship between crucian carp relative body depth (PC1) and (a) predation category, (b) maximum predator gape size (cm), (c) crucian carp proportion (%), and (d) trophic position

The range of littoral reliance and trophic position values observed in each population was significantly different among predation categories (PERMANOVA, Pseudo‐*F*: 27.35, *p*‐value: 0.001) and lakes (PERMANOVA, Pseudo‐*F*: 42.67, *p*‐value: 0.001). However, significant differences may be caused by different dispersion of isotopic values for both predation categories (PERMIDISP, Pseudo‐*F*: 10.45, *p*‐value: 0.001) and lakes (PERMIDISP, Pseudo‐*F*: 9.23, *p*‐value: 0.001), suggesting great variation in individual resource use within assemblages. Results of model selection for crucian carp body depth show that predator maximum gape size, individual trophic position, and crucian carp size were the best variables explaining variation among predation categories (Tables 4 and 5, Figure 5). It is to be noticed that maximum gape size was also negatively correlated with crucian carp density. Littoral reliance, total phosphorus, predator proportion, and lake depth were excluded from the final model during model selection.

**TABLE 4:** Model selection for body height of crucian carp with biotic and abiotic environmental parameters as explanatory variables: predator proportion (RelPred), maximum gape size (MaxGS), trophic position (TP), littoral reliance (LIT), total phosphorus (TotP), maximum depth (MaxD), and body size (logCsize)

Model	AIC	ΔAIC	Wi
*PC1 ~ MaxGS + TP + logCsize*	−2,324.98	0.00	0.982
*PC1 ~ MaxGS + TP + TotP + logCsize*	−2,316.90	8.08	0.017
*PC1 ~ RelPred + MaxGS + TP + TotP + logCsize*	−2,308.73	16.25	0.000
*PC1 ~ RelPred + MaxGS + TP + TotP + MaxD + logCsize*	2,298.44	26.54	0.000
*PC1 ~ RelPred + MaxGS + TP + LIT + TotP + MaxD + logCsize*	−2,283.78	41.20	0.000

Note

AIC, difference in AIC (ΔAIC) and Akaike weights (Wi) for candidate models are shown.

**TABLE 5:** Results of the best linear mixed model (*PC1 ~ MaxGS + TP + logCsize*) explaining the relation between crucian carp body shape and maximum gape size (MaxGS), trophic position (TP), body size (logCsize)

Effect	Estimate	*SE*	*df*	*t‐value*	*p*‐value
*(Intercept)*	0.00002	0.0036	2.10	0.01	0.996
*MaxGS*	0.02700	0.0036	2.59	7.43	0.008*
*TP*	−0.00284	0.0008	354.00	−3.46	0.001*
*logCsize*	0.00289	0.0009	353.30	3.15	0.002*
*Marginal R^2^*: 0.81; *Conditional R^2^*: 0.94					

* *p*‐value < 0.05.

## 4 DISCUSSION

The body shape of crucian carp differed significantly among the lakes investigated, and this variation was given mostly by differences in relative body depth. This difference in body shape was related to a gradient of predation risk represented by the predator community of each lake, which caused progressively deeper bodies, larger size, and lower population densities. Variation in body depth was related mainly to the maximum gape size reached by the predators in the different communities and crucian carp trophic position.

In general, in absence of predators, fish were smaller and had a more slender body shape and gradually showed higher absolute and relative body depth values when trout and perch were present, reaching the largest size and deepest bodies in pike lakes. Previous field studies, in line with our findings, show that crucian carp had a deeper body depth in populations sympatric with predators compared to allopatric ones (Poléo et al., 1995), but did not test the effect of specific predator communities. Experimental studies observed the effect of single predator species under controlled conditions and showed that crucian carp increased in body depth when exposed to cues from perch or pike and that the latter induced a more pronounced development (Brönmark & Pettersson, 1994). In our study, we also observed a smaller but significant increase in body depth of crucian carp from lakes with trout as the only predator present. Indeed, laboratory experiments showed that crucian carp was able to detect detailed information from waterborne cues such as predator diet or relative size. Individuals, for instance, showed different fright responses, as a decrease in swimming activity, when exposed to cues from large or small predators, or when these were fed crucian carp or invertebrates (Pettersson et al., 2000) Moreover, crucian carp exhibited different behavioral and neural responses to skin extract from trout, perch, and pike (Lastein et al., 2012). Fish also reduced activity levels and shifted to a nocturnal activity pattern when occurring with diurnal predators such as pike (Vinterstare, Hulthén, Nilsson, Nilsson, et al., 2020). These findings suggest that crucian carp may develop specific responses in presence of certain piscivorous fish species. A similar example of flexible predator‐induced morphological defenses is represented by *Rana pirica* tadpoles, which develop a specific body shape in response to predators with different predation strategies (Kishida & Nishimura, 2005).

However, trout, perch, and pike lakes reflected a gradient in predation efficiency which was mainly defined by maximum mouth opening, making it difficult to distinguish the effect of predator community from the gape size. Still, perch in Lake Øvresetertjern and trout in the oligotrophic Lake Posttjernet reached the largest body and gape size relative to the other lakes with the same predator species. In these lakes, crucian carp had the highest body depth in relation to the other lakes from the respective predation category. Moreover, in Lakes Øvresetertjern, Svartkulp, and Posttjernet, where perch and trout made up more than half of the species present, crucian carp had a deeper body compared to the Lakes Bjørnmyrdammen, Småvanna, and Karussputten, where predators represented a smaller proportion of the total fish (14%–18%). However, in Lakes Stomperudtjernet and Nusttjennet, predators made up only a very small proportion of the total fish community (5%) and consisted mainly of few large pike and perch. Nevertheless, in these lakes, crucian carp reached the largest size and deepest body. In the presence of perch or trout, which undergo ontogenetic diet shifts to piscivory, crucian carp may grow considerably in body depth (Brönmark & Pettersson, 1994). On the other hand, pike, a largely piscivorous and highly efficient predator which share the same vegetated habitat with crucian carp, represents a constant threat. Thus, in presence of pike, crucian carp might have developed an effective adaptive response to predation risk, independently from its density. Moreover, in these lakes, predation risk might be intensified due to the presence of perch. The coexistence of perch and pike may impose a greater risk for crucian carp of different size classes both due to the greater gape size range but also to their very different foraging behavior (Eklöv & Diehl, 1994). Thus, our results support previous experimental studies suggesting that the development of a deep body represents a morphological defense against gape‐limited piscivores (Nilsson & Brönmark, 2000). In particular, body depth determines prey size refuge, decreasing substantially vulnerability to predation (Nilsson et al., 1995). Moreover, this development in body depth would stop as soon as crucian carp reach the most functional morphology, that is, the size in which it is outside of the predation window. Indeed, in an experimental setting, removal of cues from predators resulted in a decrease in crucian carp relative body depth (Brönmark & Pettersson, 1994). While reaching a certain body shape in natural conditions is not directly comparable to the removal of predator cues in the laboratory, it suggests that a high body depth might be costly to maintain and that this development would be supported only when the predation risk is certain (Pigliucci, 2005). Thus, this variation in body shape does not seem to be the result of the simple exposure to predators, but more likely it is finely tuned with the specific structure and ecology of the predator communities (Holopainen, Aho, et al., 1997; Johansson & Andersson, 2009; Pettersson & Brönmark, 1997).

Regulation of development of body shape in response to predation risk seems to be a complex process, and indirect effects such as food availability and behavioral responses can also affect fish body morphology and growth at a fine scale (Pettersson & Brönmark, 1997; Svanbäck et al., 2017). In this regard, it was proposed that predator‐induced morphological defenses are a by‐product of prey behavior, since predators can intimidate prey inducing a decrease in their foraging activity (Peacor, 2002). This reduction in movement has been hypothesized to lower prey metabolism with a reallocation of the energy saved to increased growth or development of defense structures (Bourdeau & Johansson, 2012). Other studies suggest a link between stress physiology and the expression of inducible defense traits (Middlemis Maher et al., 2013; Vinterstare et al., 2020). Our results show distinctly that progressively deeper bodies were accompanied by an overall increase in fish size. Moreover, crucian carp density was decreasing with increasing predation risk, which may have resulted in more available resources for surviving individuals. In presence of efficient predators such as pike, few large and high‐bodied crucian carp were present. On the opposite, small‐sized individuals occurred in higher densities in absence of predators. In this regard, piscivorous fish can affect the structure of prey communities and indirectly regulate resource availability through size‐selective predation (Heynen et al., 2017). Predation can reduce prey density through direct consumption of small individuals, causing competitive release and eventually leading to an increase in somatic growth of survivors (Craig et al., 2006; Persson et al., 1996; Svanbäck & Persson, 2004). In contrast, dense populations in allopatric lakes have to compete for resource and their body condition remains low. This also suggests a potential higher growth rate with increasing predation risk (Vøllestad et al., 2004). Lake productivity also plays an important role in these dynamics, since it regulates resource availability and ultimately population density and somatic growth (Weber et al., 2010). Previous studies show that crucian carp achieved a deep body in a few months if low densities of shallow‐bodied fish were introduced into a food‐rich environment without piscivores (Holopainen, Aho, et al., 1997). However, discerning between the effects of predation and food availability is difficult in the present study, since the most productive lakes corresponded greatly to the ones with pike as main predator, making it difficult to isolate the two different effects. Remarkably, crucian carp from the allopatric pond Forkerudtjern, one of the most productive among the study systems, had the highest relative body depth respect to the other lakes with no predators, but fish were still considerably stunted, probably because of the high population density.

Individual resource use did not have a strong direct effect on crucian carp body shape. Trophic ecology of crucian carp was different among lakes, but our results do not show a clear shift in resource use induced by predation risk. Generally, fish seemed to rely on littoral invertebrates associated with substrate or vegetation, but at the same time, individual resource use varied greatly within each lake. A possible reason for the lack of correlation between body shape and resource use might be that many of the fish caught were already outside of the predation window and thus probably able to forage more actively and exploit different food resources. Moreover, fish might be able to easily use the resources from both the pelagic and littoral habitat since the study lakes were mostly small and both habitats are next to each other (Scharnweber et al., 2013). Furthermore, we did not catch any fish from the profundal habitat of deeper lakes—that is, maximum depth of around 11 m—suggesting that crucian carp were still confined to the shallow area. In contrast, crucian carp body depth was related to trophic position, and, in particular, different predator communities seemed to have specific effects. Trophic position had a positive influence on body depth in allopatric and trout lakes. With absent or low predation risk, one of the main limiting factors for crucian carp to feed on different resources could be mouth gape, as fish are able to exploit larger sized invertebrate resources only when they reach a certain body depth or size. In pike lakes, trophic position was slightly lower. Here, crucian carp hiding in the vegetation might feed on macrophytes and large invertebrates such as snails and clams, which likely lowers the trophic position when compared to zooplankton feeding. This also corresponds with a lowering of crucian carp activity, as fish expend less energy in foraging.

Crucian carp body depth increased along a gradient of predation risk represented by increasingly efficient predator categories. Specifically, our results indicate that crucian carp is provided with a fine‐tuned morphological defense mechanism against gape‐limited piscivores. The mechanism that triggers and regulates a change in body shape does not seem to be solely regulated by exposure to predators (Brönmark & Pettersson, 1994; Durajczyk & Stabell, 2014), but also depends on the specific structure and ecology of the predator communities. In many natural systems, prey organisms experience complex predation regimes. Species composition and abundance of predators can vary over time, especially in small lakes that are characterized by frequent fish mortality during winter (e.g., Lappalainen et al., 2016). In these small systems with high environmental stochasticity, plastic responses are advantageous since organisms are likely to be subject to strong interannual variability of predation pressure rather than constant predation risk (Kishida & Nishimura, 2006). Under such conditions, seasonal and annual changes in resource availability are also likely to occur, and generalist feeding strategies might be favored over specialization in acquiring specific resources (Scharnweber et al., 2013). Crucian carp flexibility in niche use is complex and needs to be better explored considering not only predation pressure, but also the competitive interactions and the abiotic conditions shaping these systems. In this sense, a limitation of this study was that the gradient of predation pressure corresponded to a shift in lake productivity and fish community, making it difficult to discern between the effects of predation risk and other environmental factors. For example, pike lakes were the most productive and had a complex fish community respect to the trout and perch lakes. However, this is an intrinsic characteristic of these systems, and crucian carp variation in body shape is likely a result of different ecological processes which act in synergy with specific predation risk. Moreover, though laboratory and field experiments show that this change in crucian carp body shape is mainly due to a plastic response, it could also be related to evolutionary responses, that is, natural populations may present differences in both their phenotypes and the extent of plasticity of those phenotypes as the product of natural selection within each population. Thus, further field and experimental studies should investigate if there is a genomic component to observed phenotypic differentiation.

## CONFLICT OF INTEREST

Authors have no conflict of interest to declare.

## AUTHOR CONTRIBUTION

**Ilaria de Meo:** Conceptualization (lead); data curation (lead); formal analysis (lead); investigation (lead); methodology (lead); project administration (equal); supervision (lead); writing – original draft (lead). **Kjartan Østbye:** Conceptualization (equal); methodology (equal); resources (equal); supervision (equal); writing‐review & editing (supporting). **Kimmo K. Kahilainen:** Conceptualization (equal); formal analysis (supporting); methodology (equal); supervision (equal); writing‐review & editing (supporting). **Brian Hayden:** Conceptualization (supporting); formal analysis (supporting); resources (equal); supervision (supporting); writing‐review & editing (supporting). **Christian H. H. Fagertun:** Conceptualization (supporting); investigation (equal); writing‐review & editing (supporting). **Antonio B. S. Poleo:** Conceptualization (equal); project administration (lead); resources (lead); supervision (equal); writing‐review & editing (supporting).

## Supporting information

Supplementary Material

## Data Availability

Landmark coordinates, stable isotope values, and environmental data used in this study are available on Dryad at https://doi.org/10.5061/dryad.d2547d825.
