# Supplementary material for: Predator community and resource use jointly modulate the inducible defense response in body height of crucian carp
Source: Ecol Evol. 2021 Feb 3;11(5):2072–85. doi: 10.1002/ece3.7176 (PMC7920785; doi:10.1002/ece3.7176)
Supplement: Supplementary file 1 — Supplementary Material [file ECE3-11-2072-s001.pdf]

SUPPORTING INFORMATION

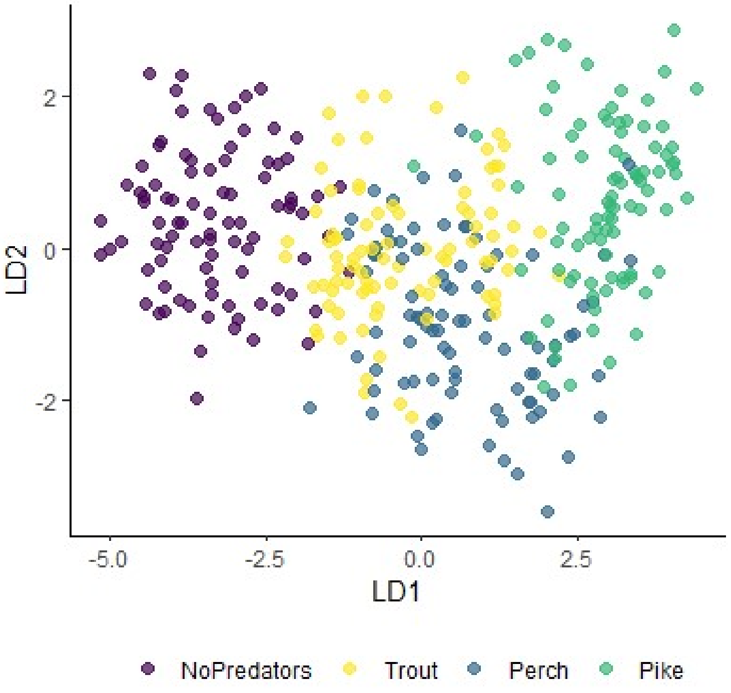

Figure S1: Scatterplot of discriminant function analysis of body shape of crucian carp grouped according to predation category.

Table S1: Contribution of landmark coordinates to the first three axes of the principal components analysis (PCA). Landmarks are indicated in Figure 2.

	Comp1	***Comp2***	***Comp3***
1.X	0.231	0.071	0.300
1.Y	-0.022	0.248	0.027
2.X	0.102	0.030	0.029
2.Y	-0.044	-0.022	-0.180
3.X	-0.138	0.152	-0.109
3.Y	-0.091	-0.086	-0.222
4.X	-0.123	-0.110	-0.192
4.Y	0.206	-0.392	-0.175
5.X	-0.095	0.020	-0.207
5.Y	0.494	-0.407	-0.071
6.X	0.203	-0.071	0.018
6.Y	0.169	-0.139	0.110
7.X	-0.133	-0.125	0.297
7.Y	0.112	0.186	-0.060
8.X	-0.031	0.027	0.338
8.Y	0.039	0.216	-0.103
9.X	-0.099	0.143	0.102
9.Y	-0.009	0.229	-0.118
10.X	0.052	0.160	-0.083
10.Y	-0.089	0.002	0.027
11.X	0.013	0.186	-0.175
11.Y	-0.268	-0.075	0.139
12.X	-0.248	-0.061	0.033
12.Y	-0.343	-0.271	0.276
13.X	-0.095	-0.126	-0.252
13.Y	-0.159	-0.136	0.206
14.X	-0.104	-0.136	0.081
14.Y	0.007	0.028	0.123
15.X	0.110	0.021	-0.004
15.Y	-0.013	0.072	0.165
16.X	0.164	-0.022	0.179
16.Y	0.067	0.220	0.058
17.X	0.185	0.064	0.075
17.Y	-0.011	0.098	-0.025
18.X	0.104	0.034	0.027
18.Y	-0.083	0.065	-0.092
19.X	-0.011	-0.014	-0.070
19.Y	-0.013	0.092	-0.048
20.X	0.104	0.024	0.010
20.Y	0.104	0.142	0.038
21.X	0.104	0.027	0.019
21.Y	-0.008	0.098	-0.030
22.X	-0.106	-0.096	-0.203
22.Y	-0.021	-0.040	-0.088
23.X	-0.188	-0.197	-0.211
23.Y	-0.024	-0.127	0.044

Table S2: Jack-knifed classification results of crucian carp body shape from discriminant function analysis (DFA). Values in bold indicate the number of individuals correctly classified into each predation category.

	% Correct:	***No Predators***	***Trout***	***Perch***	***Pike***	***Total n***
No Predators	92%	**83**	7	0	0	90
Trout	69%	3	**62**	17	8	90
Perch	64%	0	26	**58**	6	90
Pike	90%	0	2	7	**81**	90

Table S3. Results of Procrustes ANOVA of crucian carp body shape in relation to centroid size (i.e. logCsize), predation category (Pred), interaction (logCsize : Pred) and interaction with lake (logCsize : Pred: Lake). '*': *P-value* < 0.05.

**Effect**	**df**	**SS**	**MS**	R^2	**F**	**Z**	**P-value**
**Log (Csize)**	1	0.210	0.210	0.255	191.423	8.620	0.001 *
**Pred**	3	0.153	0.051	0.190	46.414	11.662	0.001 *
**Log (Csize) : Pred**	3	0.024	0.008	0.029	7.192	7.692	0.001 *
**Log (Csize) : Pred : Lake**	8	0.061	0.008	0.073	6.891	11.482	0.001 *

df, degrees of freedom; SS, sums-of-squares; MS, mean square; F, F statistic; Z, effect sizes.
